# Colloidal Antimony Sulfide Nanoparticles as a High-Performance Anode Material for Li-ion and Na-ion Batteries

**DOI:** 10.1038/s41598-020-59512-3

**Published:** 2020-02-13

**Authors:** Kostiantyn V. Kravchyk, Maksym V. Kovalenko, Maryna I. Bodnarchuk

**Affiliations:** 1 Laboratory for Thin Films and Photovoltaics, Empa – Swiss Federal Laboratories for Materials Science and Technology, Überlandstrasse 129, CH-8600 Dübendorf, Switzerland; 2 Laboratory of Inorganic Chemistry, Department of Chemistry and Applied Biosciences, ETH Zürich, Vladimir-Prelog-Weg 1, CH-8093 Zürich, Switzerland

**Keywords:** Energy science and technology, Energy storage, Batteries

## Abstract

To maximize the anodic charge storage capacity of Li-ion and Na-ion batteries (LIBs and SIBs, respectively), the conversion–alloying-type Sb_2_S_3_ anode has attracted considerable interest because of its merits of a high theoretical capacity of 946 mAh g^−1^ and a suitable anodic lithiation/delithiation voltage window of 0.1–2 V *vs*. Li^+^/Li. Recent advances in nanostructuring of the Sb_2_S_3_ anode provide an effective way of mitigating the challenges of structure conversion and volume expansion upon lithiation/sodiation that severely hinder the Sb_2_S_3_ cycling stability. In this context, we report uniformly sized colloidal Sb_2_S_3_ nanoparticles (NPs) as a model Sb_2_S_3_ anode material for LIBs and SIBs to investigate the effect of the primary particle size on the electrochemical performance of the Sb_2_S_3_ anode. We found that compared with microcrystalline Sb_2_S_3_, smaller *ca*. 20–25 nm and *ca*. 180–200 nm Sb_2_S_3_ NPs exhibit enhanced cycling stability as anode materials in both rechargeable LIBs and SIBs. Importantly, for the *ca*. 20–25 nm Sb_2_S_3_ NPs, a high initial Li-ion storage capacity of 742 mAh g^−1^ was achieved at a current density of 2.4 A g^−1^. At least 55% of this capacity was retained after 1200 cycles, which is among the most stable performance Sb_2_S_3_ anodes for LIBs.

## Introduction

Lithium-ion batteries (LIBs) are the most well-known rechargeable electrochemical energy storage devices, and they are a key component of electric mobility and portable electronics^1–4^. Sodium-ion batteries (SIBs) are conceptually similar, and they have attracted enormous attention in recent years because of the higher natural abundance of sodium and more favorable distribution of sodium reserves compared with lithium^5–13^. Although graphite is currently the main commercialized anode material for LIBs, its low theoretical charge storage capacity (372 mAh g^−1^) limits its application in new generation batteries, requiring exploration of new electrode materials with higher capacity and stable cycling performance^14^. With respect to SIBs, the search for efficient Na-storing anodes is a high priority, because graphite shows low Na-ion capacities of 30–35 mAh g^−1^^15^, while other carbonaceous materials have low tap densities and exhibit capacities of less than 300 mAh g^−1^^16^. Additionally, the relatively low potential of carbon sodiation (~0 V *vs*. Na^+^/Na) leads to deposition of sodium metal on the carbon electrode surfaces, which may eventually result in compromised safety^17^.

Over the past decade, much attention has been focused on development of alternative anode materials for both LIBs and SIBs^12^. In particular, low-cost and environmentally benign Sb_2_S_3_ anodes have attracted great interest because of their high capacities and relatively low redox lithiation/sodiation potentials^18–45^. Theoretically, Sb_2_S_3_ can generate a specific capacity as high as 946 mAh g^−1^ through conversion and alloying reactions (corresponding to 12 mol of lithium/sodium and electrons per formula unit). However, harnessing this storage potential of Sb_2_S_3_ is hindered by its poor capacity retention owing to the structural (conversion) and volume (alloying) changes during discharging/charging, which lead to mechanical disintegration of the electrodes and thus loss of electrical connectivity. These difficulties can be mitigated by nanostructuring, particularly when the active material is embedded in an elastic and conductive network that helps to enhance electronic transport and reduce the cycling instability caused by volumetric changes in the conversion–alloying-type anode material^36,46,47^. Specifically, in the last few years, extensive effort has focused on various forms of nanostructured Sb_2_S_3_, such as Sb_2_S_3_ nanowires^24,48–51^, nanorods^45,52,53^, nanoparticles (NPs)^37,54–57^, nanocables^58^, and Sb_2_S_3_/C nanocomposites^23^, to maximize the anodic charge-storage capacity and improve the cycling performance. Notably, the electrochemical performance of highly uniform colloidal Sb_2_S_3_ NPs has not been reported. Such NPs are an ideal platform for studying the effects of the size and electrode morphology on the charge storage capacity and cycling stability of Sb_2_S_3_ anodes.

In this study, we synthesized uniformly sized colloidal Sb_2_S_3_ NPs whose size is tunable in 10–200 nm size range, which allowed us to comprehensively investigate the effect of the primary particle size on the electrochemical behaviour of Sb_2_S_3_ as the anode material for LIBs and SIBs. We assessed the pros and cons of nano-Sb_2_S_3_ anodes in comparison with commercial microcrystalline Sb_2_S_3_ (hereafter denoted bulk Sb_2_S_3_, Figure S1). We note that although synthesis of Sb_2_S_3_ NPs might be prohibitively expensive for practical application in commercial batteries, the insight gained from using such precisely tunable model NPs can guide development of Sb_2_S_3_ anodes for both LIBs and SIBs. We found that at current rates of 0.3–12 A g^−1^, the Li-ion storage capacities for anodes composed of both *ca*. 20–25 nm (1055–608 mAh g^−1^) and *ca*. 180–200 nm Sb_2_S_3_ (970–574 mAh g^−1^) were significantly higher than for their bulk counterpart (683–418 mAh g^−1^). For Na-ion storage, the capacities of nano-Sb_2_S_3_ and bulk Sb_2_S_3_ anodes were similar. Regarding the cycling stability, the major finding was that nano-Sb_2_S_3_ exhibited significantly higher capacity retention for both Li-ion and Na-ion storage than bulk Sb_2_S_3_. Notably, unprecedented Li-ion capacity retention of 55% was achieved for *ca*. 20–25 nm Sb_2_S_3_ NPs at a current density of 2.4 A g^−1^ after 1200 cycles.

## Results and Discussion

**Figure 1:**
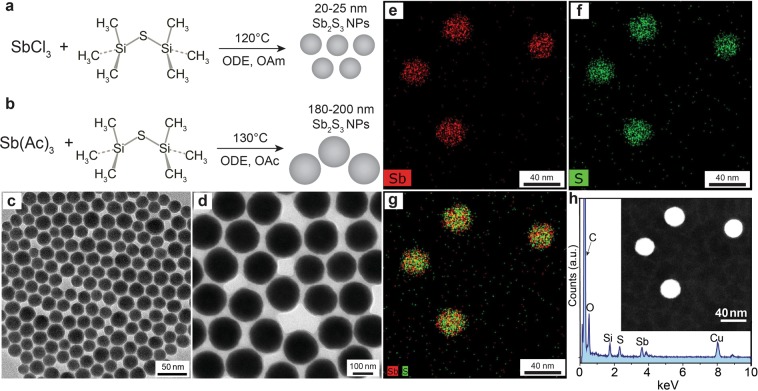
Schematics of one-pot synthesis of the (**a**) *ca*. 20–25 nm and (**b**) 180–200 nm Sb_2_S_3_ NPs. TEM images of the (**c**) *ca*. 20–25 nm and (**d**) *ca*. 180–200 nm Sb_2_S_3_ NPs. (**e**) Sb-Lα and (**f**) S-Kα elemental STEM-EDXS maps of the *ca*. 20–25 nm Sb_2_S_3_ NPs. (**g**) Reconstructed overlay image of the elemental maps shown in (**e**) and (**f**). (**h**) EDXS spectrum of the small Sb_2_S_3_ NPs. The insert shows a high-angle annular dark-field scanning transmission electron microscopy image of the *ca*. 20–25 nm Sb_2_S_3_ NPs.

The general synthetic route for preparation of *ca*. 20–25 nm amorphous antimony sulfide NPs using octadecene (ODE) as a solvent in the presence of oleylamine (OAm) as a surface capping ligand is shown in Fig. 1a. In a typical synthesis, the Sb_2_S_3_ NPs were synthesized by the hot-injection technique using antimony(III) chloride and bis(trimethylsilyl)sulfide ((TMS)_2_S) as the antimony and sulfur precursors, respectively. After injection of (TMS)_2_S into the SbCl_3_/ODE mixture, the color of the reaction solution rapidly changed to red-orange. The reaction temperature was maintained at 120 °C for 15 min. Transmission electron microscopy (TEM) and X-ray diffraction (XRD) analysis confirmed formation of amorphous spherical *ca*. 20–25 nm antimony sulfide NPs with a narrow size distribution (Figs. 1c and S2a). A longer reaction time of 30 min resulted in formation of *ca*. 1.5–2.0 μm long crystalline Sb_2_S_3_ nanorods with diameters of *ca*. 150–200 nm (Figure S3). When the (TMS)_2_S solution was injected at 100 °C and maintained at this temperature for 15 min, *ca*. 8–10 nm amorphous Sb_2_S_3_ NPs were obtained (Figure S4). Scanning transmission electron microscopy with energy-dispersive X-ray spectroscopy (STEM-EDXS) measurements of the as-synthesized *ca*. 20–25 nm Sb_2_S_3_ NPs revealed that Sb and S were homogeneously distributed throughout each NP (Fig. 1e,f, g, h). From scanning electron microscopy with energy-dispersive X-ray spectroscopy (SEM-EDXS) analysis, the atomic ratio of Sb, S and O was about 1:1.4:0.1 (Figure S5). The presence of detectable oxygen in the EDS spectrum could be because of oxidation of the NPs during synthesis, cleaning, or preparation of the specimen. Notably, similar synthesis of Sb_2_S_3_ NPs was reported by Bakr *et al*.^59^. using SbCl_3_ and (TMS)_2_S in ODE with oleic acid (OAc) as a ligand. The synthesis yielded relatively polydisperse *ca*. 30–50 nm Sb_2_S_3_ NPs with a chain-like structure. In our synthesis, the use of the OAm ligand resulted in slower reaction kinetics, causing more homogenous nucleation and growth of Sb_2_S_3_ NPs in comparison with the OAc ligand.

Larger Sb_2_S_3_ NPs of approximately *ca*. 180–200 nm were synthesized in a similar way to the *ca*. 20–25 nm Sb_2_S_3_ NPs by replacing the antimony(III) chloride precursor and OAm ligand with antimony acetate and OAc, respectively (Figs. 1b,d and S2b, S6; for details see the experimental section). By changing of the (TMS)_2_S sulfur source to S/OAm (elemental sulfur dissolved in OAm), crystalline Sb_2_S_3_ nanoplates were obtained (Figure S7).

**Figure 2:**
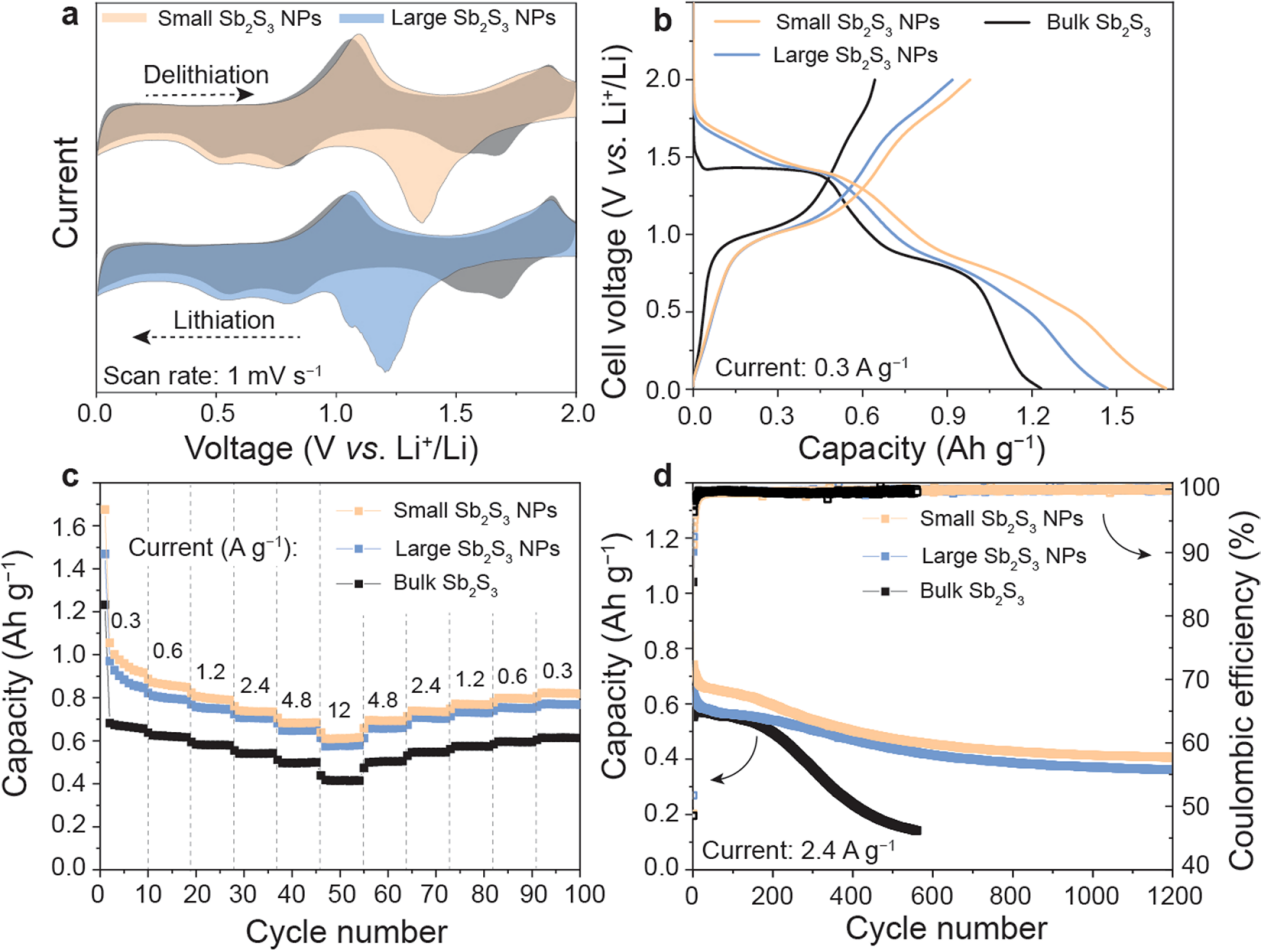
Electrochemical results of the Sb_2_S_3_ electrodes cycled with lithium electrolyte (1 M LiPF_6_ in ethylene carbonate/dimethyl carbonates (EC/DMC)) in a half-cell configuration using metallic lithium as the counter and reference electrode. (**a**) CV curves (the first cycle is shown in orange or blue and the second cycle is shown in grey) of the small and large Sb_2_S_3_ NPs measured at a scan rate of 1 mV s^−1^ (see Figure S8 for details). (**b**) Galvanostatic charge–discharge curves of the small Sb_2_S_3_ NPs, large Sb_2_S_3_ NPs, and bulk Sb_2_S_3_ during the first cycle. (**c**) Rate capacity and (**d**) cycling stability measurements of Li-ion half-cells using Sb_2_S_3_ anodes made from small Sb_2_S_3_ NPs, large Sb_2_S_3_ NPs, and bulk Sb_2_S_3_. The corresponding galvanostatic charge–discharge curves and Coulombic efficiency measured at current densities of 0.3–12 A g^−1^ and different cycle number are shown in Figures S9, S10 and S11 respectively.

**Figure 3:**
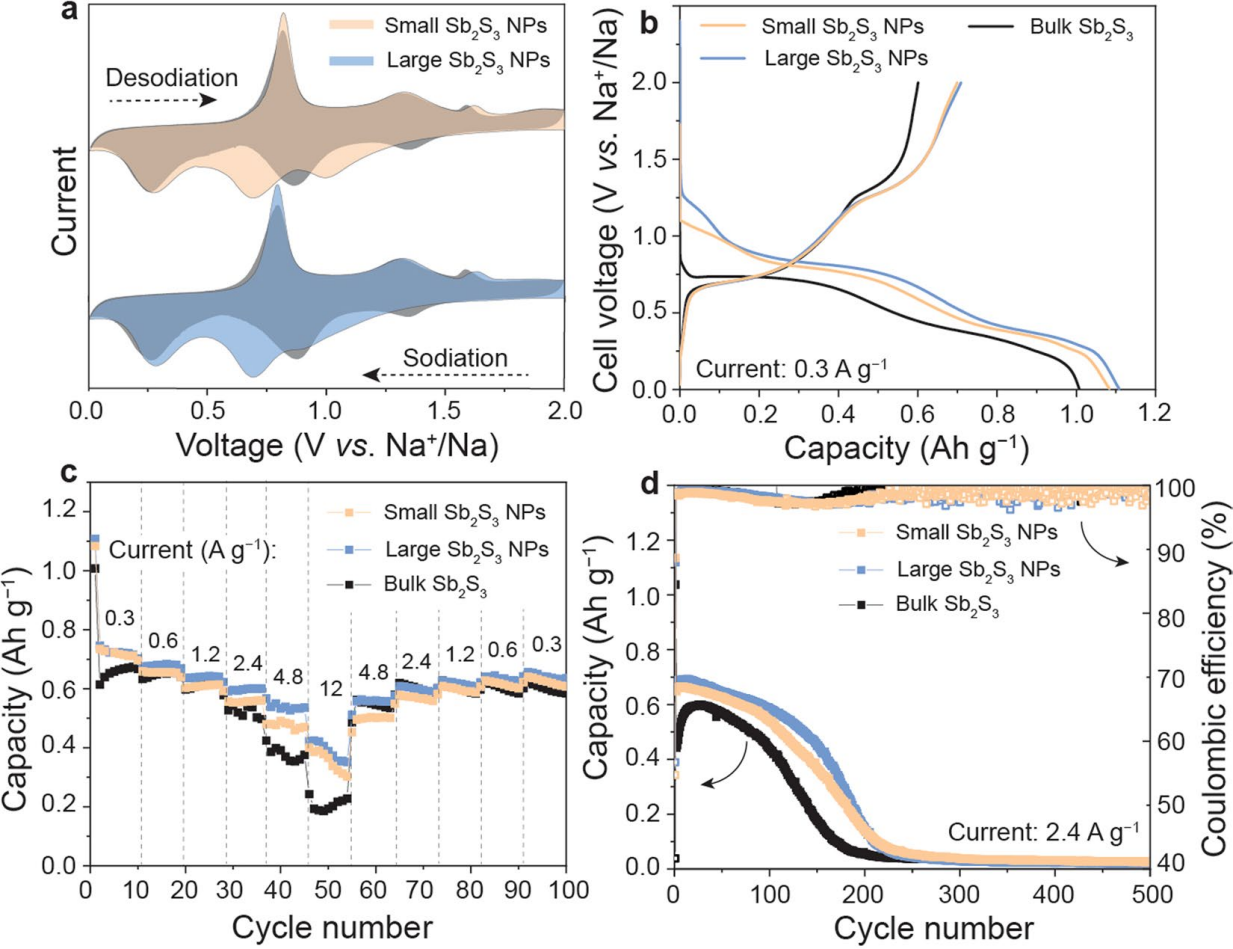
Electrochemical results of the Sb_2_S_3_ electrodes cycled with sodium electrolyte (1 M NaClO_4_ in PC) in a half-cell configuration using metallic sodium as the counter and reference electrode. (**a**) CV curves (the first cycle is shown in orange or blue and the second cycle is shown in grey) of the electrodes composed of small and large Sb_2_S_3_ NPs measured at a scan rate of 1 mV s^−1^ (see Figure S12 for details). (**b**) Galvanostatic charge–discharge curves of the electrodes composed of small Sb_2_S_3_ NPs, large Sb_2_S_3_ NPs and bulk Sb_2_S_3_ during the first cycle. (**c**) Rate capacity and (**d**) cycling stability of Na-ion half-cells using Sb_2_S_3_ anodes composed of small Sb_2_S_3_ NPs, large Sb_2_S_3_ NPs, and bulk Sb_2_S_3._ The corresponding galvanostatic charge–discharge curves and Coulombic efficiency measured at current densities of 0.3–12 A g^−1^ and different cycle numbers are shown in Figures S13, S14 and S15, respectively.

The galvanostatic cycling measurements of the Sb_2_S_3_ NPs are summarized in Figs. 2 and 3. For electrochemical testing, the Sb_2_S_3_ NPs were treated with a 1 M solution of hydrazine in acetonitrile for 2 h^60,61^. The untreated NPs gave no operational electrodes because of the isolating long-chain capping molecules surrounding the as-synthesized Sb_2_S_3_ NPs. In addition to the effect of the active material, the charge storage capacity of the electrode strongly depends on the electrode formulation (the origin and amounts of the binder and conductive additive), electrode thickness, porosity, temperature, electrolyte, and so forth. Therefore, with the aim of distinguishing the size effect from the other factors, the following experimental parameters were fixed for all of the electrodes: (i) the choice and mass fractions of the binder and carbon black and (ii) the electrolyte composition. All of the electrodes contained 64 wt% of the active material, 15 wt% carboxymethylcellulose as a binder, and 21 wt% carbon black as a conductive additive. The electrochemical tests were performed in Li-ion or Na-ion half-cells with elemental lithium or sodium acting as both the counter and reference electrodes, respectively. Further details of electrode preparation and assembly of the batteries are given in the Supporting Information.

The cyclic voltammetry (CV) curves of electrodes composed of *ca*. 20–25 nm and *ca*. 180–200 nm Sb_2_S_3_ NPs (hereafter denoted small and large Sb_2_S_3_ NPs, respectively) measured in Li-ion electrolyte at a scan rate of 1 mV s^−1^ are shown in Fig. 2a. In the first cathodic cycle, the broad peak at about 1.2–1.4 V *vs*. Li^+^/Li can be attributed to formation of a solid electrolyte interphase (SEI) layer and the conversion reaction of Sb_2_S_3_ NPs (Sb_2_S_3_ + 6Li^+^ + 6e^−^ → 2Sb + 3Li_2_S). Upon further lithiation, two reduction peaks at 0.7 and 0.5 V *vs*. Li^+^/Li appeared, which are ascribed to formation of Li_2_Sb and Li_3_Sb alloys, respectively. In the reverse scan, the Sb_2_S_3_ electrode showed two peaks at 1 and 1.9 V, which are associated with delithiation of the Li_3_Sb alloy phase following formation of Sb_2_S_3_. The discharge voltage profiles of the Sb_2_S_3_ NPs are shown in Fig. 2b. The profiles of the Sb_2_S_3_ NPs are similar to the CV curves, showing two-step reduction of Sb_2_S_3_ eventually resulting in formation of metallic Sb (conversion reaction, voltage range 1.7–1.2 V *vs*. Li^+^/Li) and the Li_3_Sb alloy (alloying reaction, voltage range 0.4–1.0 V *vs*. Li^+^/Li). As follows from CV measurements, alloying of Sb in bulk Sb_2_S_3_, large and small Sb_2_S_3_ NPs takes place differently. In the bulk system, it appears that the lithiation proceeds through the direct formation of Li_3_Sb alloy. On the contrary, in the case of Sb_2_S_3_ NPs, the lithiation takes place through sequential formation of Li_2_Sb and Li_3_Sb alloys, respectively.

The Li-ion discharge capacities of Sb_2_S_3_ anodes composed of small Sb_2_S_3_ NPs, large Sb_2_S_3_ NPs, and microcrystalline Sb_2_S_3_ at charge/discharge current densities of 0.3–12 A g^−1^ are shown in Fig. 2c. At a low current density of 0.3 A g^−1^, the anodes composed of small and large Sb_2_S_3_ NPs exhibited theoretical capacities of about 1000 mAh g^−1^ with Coulombic efficiency of 97%–98% (Figure S10). The capacity retention values of the Sb_2_S_3_ anodes composed of small and large Sb_2_S_3_ NPs were 60% and 61% at 12 A g^−1^, respectively. The slightly higher discharge capacity of the anode composed of small Sb_2_S_3_ NPs during the first few cycles at a low current density of 0.3 A g^−1^ can be attributed to formation and stabilization of a SEI layer. For the bulk Sb_2_S_3_ system, the anode composed of microparticles of Sb_2_S_3_ exhibited only 60% of the theoretical capacity at 0.3 A g^−1^, but it retained 57% of its initial charge-storage capacity at high current density, similar to the Sb_2_S_3_ NP anodes. Regarding the cycling performance, the Sb_2_S_3_ NP and bulk Sb_2_S_3_ anodes showed stable capacities for the first 200 cycles (Fig. 2d). However, upon prolonged cycling, the capacity of the bulk Sb_2_S_3_ anode gradually decreased.

The capacities of the anodes composed of small and large Sb_2_S_3_ NPs were stable for 1200 cycles. The anode composed of small Sb_2_S_3_ NPs systematically showed at least 5% higher capacity than the anode composed of large NPs. In all cases, the Coulombic efficiency was relatively low for the initial 10–20 cycles (95%–97%), but it then increased to more than 99% upon cycling. As mentioned above, the higher cycling stability of the anode composed of Sb_2_S_3_ NPs compared with that composed of bulk Sb_2_S_3_ probably originates from the lower kinetic constraints of nanomaterials for conversion and alloying reactions. For instance, for alloying anode materials (e.g., Sn, Si, and Ge), several studies have demonstrated the existence of a critical size of the particles below which they do not fracture^62,63^. Furthermore, we speculate that the amorphicity of the Sb_2_S_3_ NPs aids in isotropic expansion/contraction upon their lithiation/delithiation, eventually resulting in reduction of the amount of anisotropic mechanical stress within the electrode.

In Na-ion cells with Sb_2_S_3_ NP electrodes, CV measurements showed three peaks at *ca*. 1, 0.7, and 0.27 V associated with formation of a SEI layer/intercalation of sodium ions into Sb_2_S_3_, conversion, and alloying reactions, respectively (Fig. 3a, see Figure S12 for details). Upon desodiation (reverse scan), the Sb_2_S_3_ electrode showed two peaks at 0.8 and 1.3 V *vs*. Na^+^/Na, which are associated with dealloying of Sb and reconversion of the Sb_2_S_3_ phase. The third peak at a higher potential of 1.6 V can be assigned to deinsertion of Na^+^ ions from Sb_2_S_3_. In general, the CV curves (Fig. 3a) and shape of the voltage profiles (Fig. 3b) suggest conversion and the alloying mechanism of sodiation of the Sb_2_S_3_ NPs in the voltage ranges 0.6–1 V and 0.1–0.5 V *vs*. Na^+^/Na, respectively.

In Na-ion cells, the nano-Sb_2_S_3_ and bulk Sb_2_S_3_ electrodes showed similar charge storage capacities of ~580–620 mAh g^−1^ at current densities of 0.3–1.2 A g^−1^ (Fig. 3c). The similar capacities of the nano-Sb_2_S_3_ and bulk Sb_2_S_3_ anodes in Na-ion cells can be explained by the presence of an amorphous surface oxide shell on the Sb_2_S_3_ NPs (see Figures S5 and S6 for EDS spectra). This leads to formation of Na_2_O, eventually resulting in irreversible capacity loss in the first discharge cycle. The much smaller differences among the capacities of the electrodes composed of small Sb_2_S_3_ NPs, large Sb_2_S_3_ NPs, and bulk Sb_2_S_3_ for Na-ion cells than Li-ion cells can be explained by the different properties of Li_2_O and Na_2_O. We suspect that Li_2_O acts as a relatively benign impurity covering the Sb_2_S_3_ NPs because of its high Li-ion conductivity. In contrast, Na_2_O is a much poorer Na^+^ conductor, leading to exclusion of some Sb_2_S_3_ NPs from the reversible charge/discharge storage capacity. The results of stability tests for 500 cycles at a high current density of 2.4 A g^−1^ are shown in Fig. 3d. In general, the charge storage capacities were consistently higher for nano-Sb_2_S_3_ than bulk Sb_2_S_3_, although the capacities remained stable for only about 50 and 100 cycles for bulk Sb_2_S_3_ and nano-Sb_2_S_3_, respectively.

## Conclusions

In summary, we have reported facile colloidal synthesis of highly uniform colloidal Sb_2_S_3_ NPs with mean particle sizes in the ranges *ca*. 20–25 nm and *ca*. 180–200 nm. The underlying chemistry is based on the reaction of antimony(III) chloride/acetate and (TMS)_2_S in ODE using OAm/OAc as a coordinating ligand at high temperature of 120/130 °C for small/large Sb_2_S_3_ NPs. Both the small and large Sb_2_S_3_ NPs showed electrochemical cyclic stability superior to that of bulk Sb_2_S_3_ in both LIBs and NIBs. In particular, the small NPs exhibited high retention of the capacity upon extended cycling, losing only 55% of their initial capacity over 1200 cycles at a high density of 2.4 A g^−1^.

## Methods

### Chemicals

Oleic acid (OAc, Sigma-Aldrich), oleylamine (OAm, Acros, 80–90%), octadecene (Sigma-Aldrich), octadecene (ODE, Sigma-Aldrich), antimony (III) chloride (ABCR), antimony (III) acetate (Sigma-Aldrich), bis[trimethylsilyl]sulfide (Sigma-Aldrich), chloroform and acetone were used as received.

### Synthesis of ∼20–25 nm spherical amorphous NPs

In a typical synthesis 0.5 mL oleylamine, OAm, (Acros, 80–90%) and 4 mL octadecene (ODE) were loaded into 25-mL flask and dried at 100 °C for 30 min. Then, 114 mg (0.5 mmol) SbCl_3_ were added to the flask under argon. The reaction mixture was heated up to 120 °C and 0.5 mmol bis[trimethylsilyl]sulfide (100 μL, (TMS)_2_S) in 2 mL dried ODE was then injected into the reaction flask. The color of the solution has changed to red-orange. In 15 min reaction mixture was cool down to room temperature and washed 2 times by chloroform/acetone and separated by centrifugation. After second washing step, Sb_2_S_3_ NPs were re-dispersed in oleic acid (OAc)/chloroform mixture (50 μL OAc in 2–3 mL chloroform) and stored under ambient condition. Injection of (TMS)_2_S solution at 100 °C and maintaining this temperature through the reaction for 15 min leads to formation of 8–10 nm amorphous Sb_2_S_3_ NPs. Injection of (TMS)_2_S solution at 170–180 °C and maintaining this temperature through the reaction for 3–5 min leads to formation of micrometer-sized crystalline rods (Figure S3a). Powder XRD of as-prepared NRs shows that they are highly crystalline and their XRD pattern corresponds to stibnite phase of antimony sulfide (Figure S3b). Crystalline rods could be also obtained at 120 °C in case of longer growth time. In 30 min after injection of (TMS)_2_S the orange color of the reaction mixture started to change into a gray-black.

### Synthesis of ∼180–200 nm spherical amorphous NPs

In a typical synthesis, 2.5 mL OAc, 2.5 mL ODE and 0.5 mmol antimony (III) acetate were loaded into 25-mL flask and dried at 100 °C for 30 min. The reaction mixture was heated up to 130 °C under argon. At 130 °C, 0.375 mmol (TMS)_2_S (78 μL) in 2.5 mL dried ODE was then injected into the reaction flask. The color of the solution has changed to orange. In 3–5 min, reaction mixture was cool down to room temperature and final product was washed 2 times by chloroform/acetone and separated by centrifugation. After washing Sb_2_S_3_ NPs were re-dispersed in OAc/chloroform mixture (50 μL OAc in 2–3 mL chloroform) and stored under ambient condition.

### Synthesis of thin crystalline Sb_2_S_3_ nanoplatelets

We have found that another sulfur source such as elemental sulfur in OAm effects on the morphology of Sb_2_S_3_ NPs yielding the formation of thin crystalline Sb_2_S_3_ nanoplatelets (Figure S7a). Their average size is approximately several hundred nanometers and their XRD pattern suggest that they are highly crystalline (Figure S7b). In a typical synthesis, 5 mL OAm (Acros) and 0.25 mmol (57 mg) antimony (III) chloride were loaded into 25-mL flask and dried at 80 °C for 30 min. The reaction mixture was heated up to 110 °C under argon. At 110 °C, 1 mmol (32 mg) sulfur dissolved in 2 mL OAm (Acros) was then injected into the reaction flask. Then temperature of reaction mixture was increased to 180 °C and kept for 15 min. The final product was washed 2 times by chloroform/acetone and separated by centrifugation. Sb_2_S_3_ nanoplatelets were re-dispersed in OAc/chloroform mixture (50 μL OAc in 2–3 mL chloroform) and stored under ambient condition.

### Battery components

Carbon black (Super C65, TIMCAL), carboxymethyl cellulose (CMC, Grade: 2200, Lot No. B1118282, Daicel Fine Chem Ltd.), NaClO_4_ (98%, Alfa Aesar, additionally dried), propylene carbonate (BASF, battery grade), 4-fluoro-1,3-dioxolan-2-one (FEC, Hisunny Chemical, battery grade), 1 M solution of LiPF_6_ in ethylene carbonate/dimethyl carbonate (EC/DMC, Novolyte, Celgard separator (Celgard 2400, 25 µm microporous monolayer polypropylene membrane, Celgard Inc. USA), glass microfiber separator (GF/D, Cat No. 1823–257, Whatman), Al foil (MTI Corporation), Na foil (Sigma-Aldrich), Li foil (MTI Corp.), Sb_2_S_3_ (99.995%, Sigma Aldrich), Coin-type cells (Hohsen Corp., Japan),

### Electrochemical characterization of antimony sulfide

Coin-type cells were assembled in an argon-filled glove box (O_2_ < 1 ppm, H_2_O < 1 ppm) using one layer separator (glass fiber) for NIBs and two layers of separators (Celgard and glass fiber) for LIBs. Elemental sodium or lithium served as both reference and counter electrodes. As electrolyte 1 M NaClO_4_ in PC was used for Na-ion batteries and 1 M LiPF_6_ in EC:DMC (1:1 by wt.) for Li-ion batteries. To improve cycling stability 3% of FEC were added to both electrolytes. Electrochemical measurements were performed using constant current mode for both, charge and discharge steps between 0.01–2.5 V for both Na and Li-ion batteries on a MPG2 multi-channel workstation (Bio-Logic).

### Materials characterization

TEM samples were prepared by dropping a solution of Sb_2_S_3_ NPs onto standard amorphous carbon-coated TEM grids. TEM images were recorded using JEOL JEM-2200FS microscope operated at 200 kV, STEM images and EDXS spectrum were collected on FEI Talos F200X operated at 200 kV and equipped with Super-X EDS system (4 detector configuration). Scanning electron microscopy (SEM) measurements were done on a Quanta 200 F microscope (Thermo Fisher Scientific) operated at an acceleration voltage Vacc = 20 kV. Energy-dispersive X-ray spectroscopy (EDXS) was performed with an Octane SDD detector (EDAX (Ametec)) attached to the microscope column. Powder X-ray diffraction pattern was collected with STOE STADIP powder diffractometer.

## Supplementary information

Supplementary information
