# Supplementary material for: Colloidal Antimony Sulfide Nanoparticles as a High-Performance Anode Material for Li-ion and Na-ion Batteries
Source: Sci Rep. 2020 Feb 13;10:2554. doi: 10.1038/s41598-020-59512-3 (PMC7018818; doi:10.1038/s41598-020-59512-3)
Supplement: Supplementary file 1 — Supplementary information [file 41598_2020_59512_MOESM1_ESM.docx]

*Supporting Information for*

Colloidal Antimony Sulfide Nanoparticles as a High-Performance Anode Material for Li-ion and Na-ion Batteries

Kostiantyn V. Kravchyk,^a,b*^ Maksym V. Kovalenko,^a,b^ and Maryna I. Bodnarchuk^b*^

^a^ Laboratory for Thin Films and Photovoltaics, Empa – Swiss Federal Laboratories for Materials Science and Technology, Überlandstrasse 129, CH-8600 Dübendorf, Switzerland

^b^ Laboratory of Inorganic Chemistry, Department of Chemistry and Applied Biosciences, ETH Zürich, Vladimir-Prelog-Weg 1, CH-8093 Zürich, Switzerland

*Corresponding authors

E-mail addresses: maryna.bodnarchuk@empa.ch and kravchyk@inorg.chem.ethz.ch


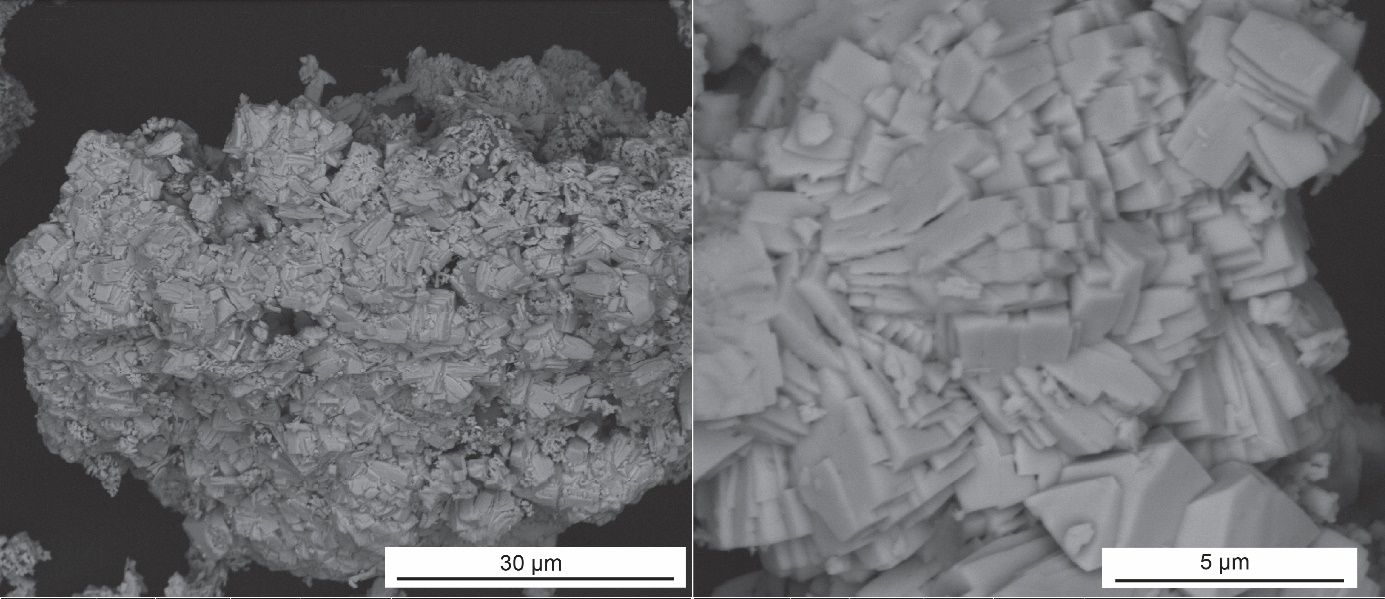


**Figure S1.** SEM images of bulk Sb_2_S_3_ particles.


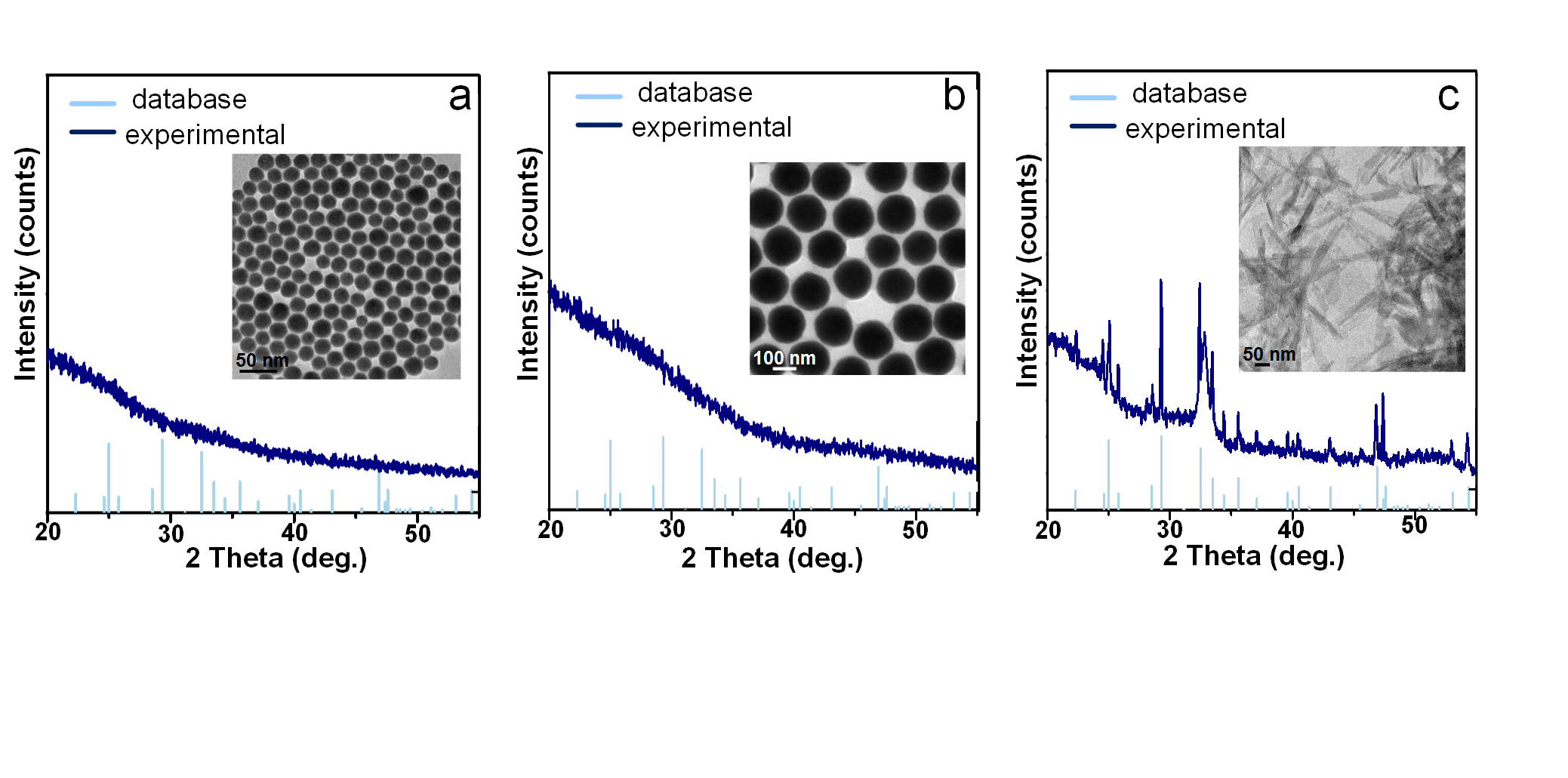


**Figure S2.** Powder X-ray diffraction pattern of *ca*. 20-25 nm (a) and *ca*. 180-200 nm (b) amorphous Sb_2_S_3_ NPs (insets: TEM images of Sb_2_S_3_ NPs).


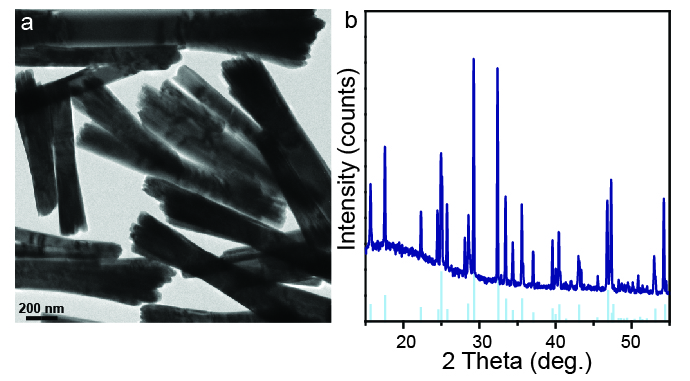


**Figure S3.** TEM image (a) and powder X-ray diffraction pattern (b) of Sb_2_S_3_ nanorods.


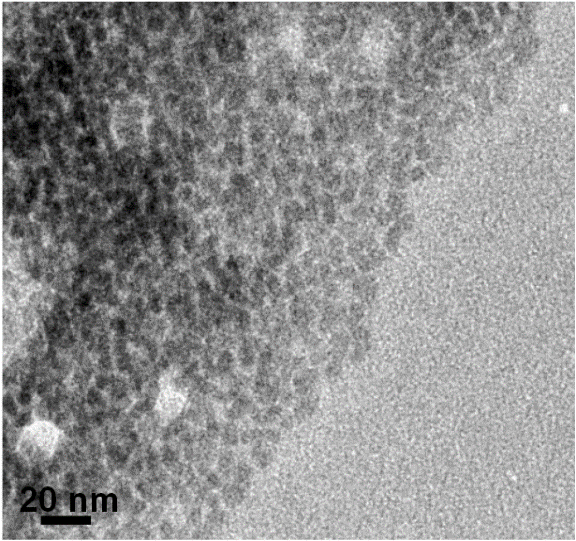


**Figure S4.** TEM image of amorphous *ca*. 8-10 nm Sb_2_S_3_ NPs obtained at 100°C.

**
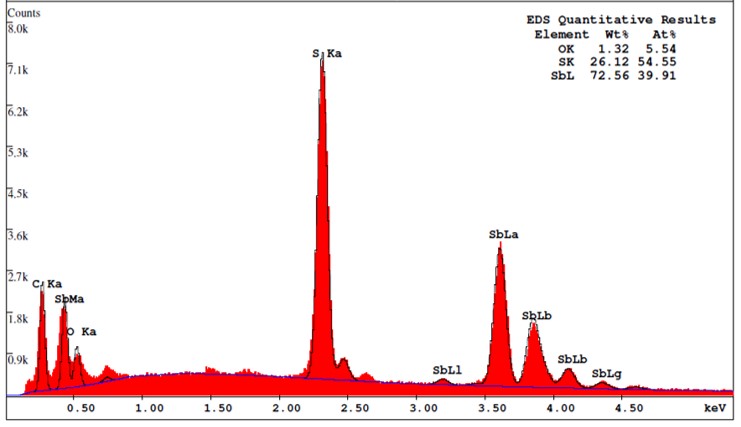
**

**Figure S5.** EDS spectra of *ca*. 20-25 nm Sb_2_S_3_ NPs.


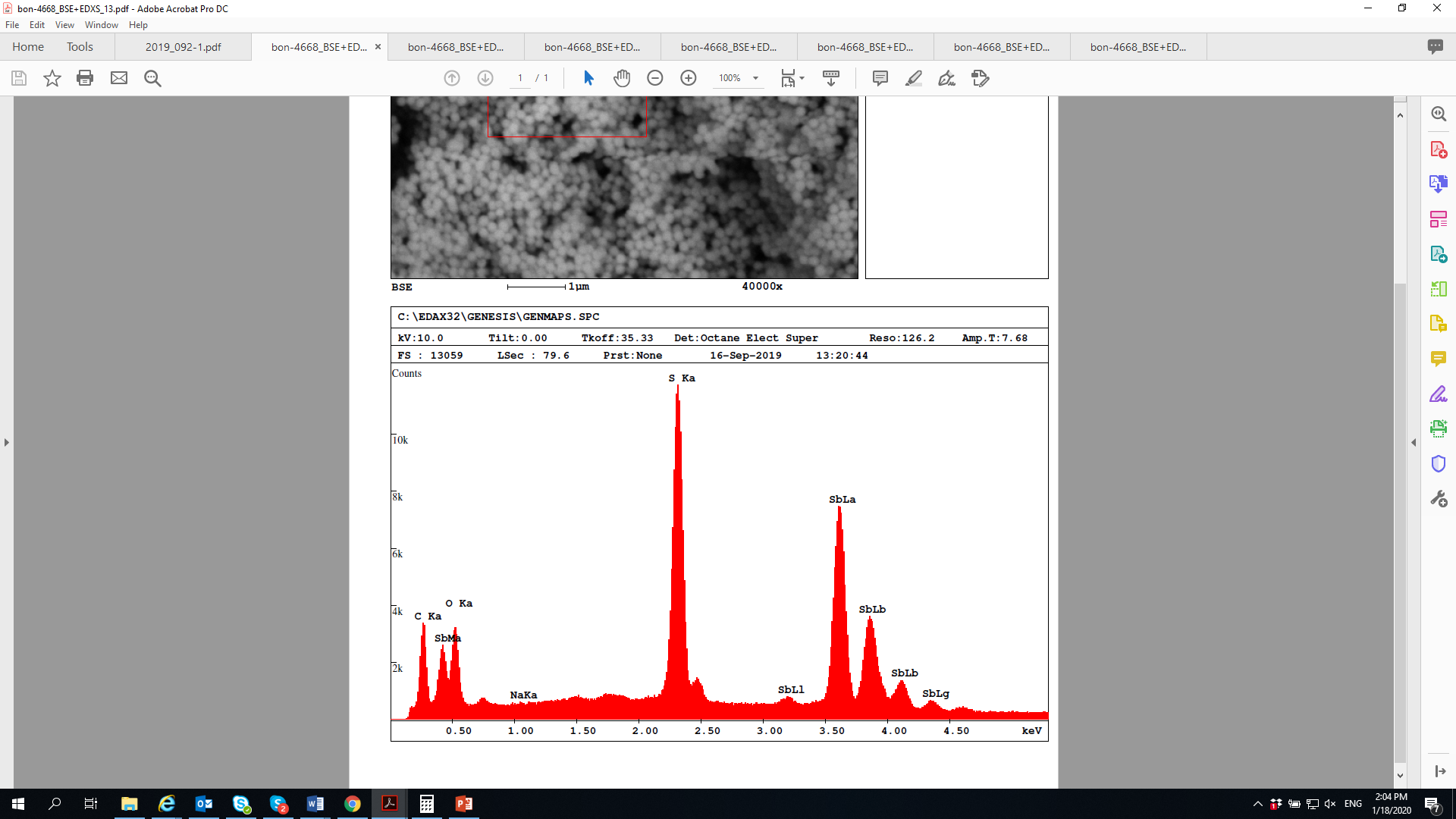

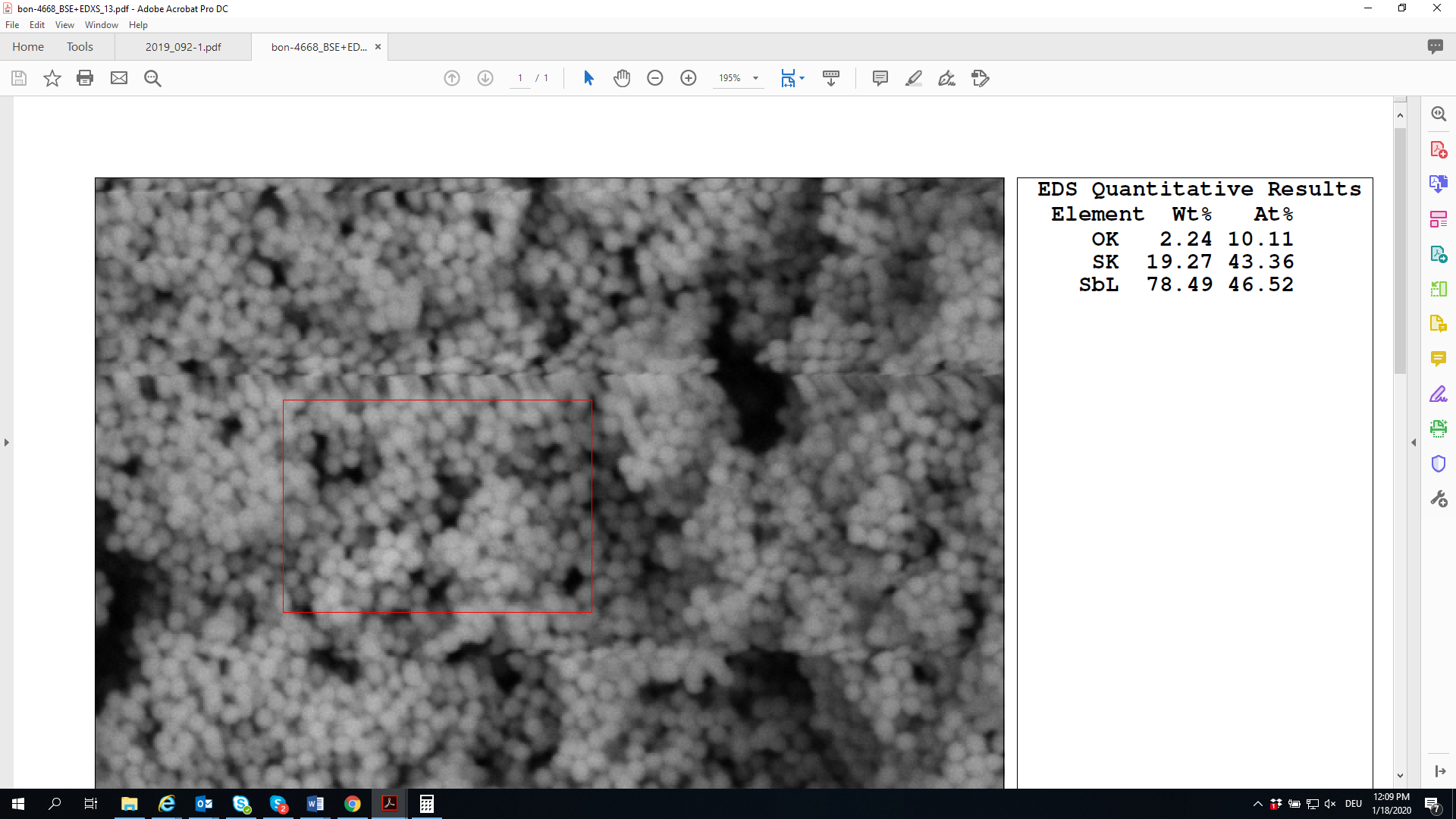


**Figure S6.** EDS spectra of *ca*. 180-200 nm Sb_2_S_3_ NPs.


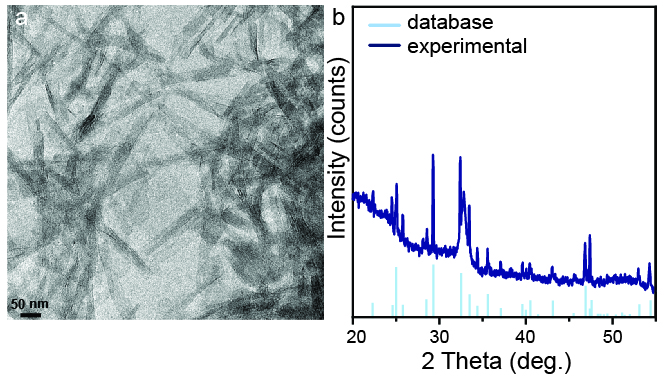


**Figure S7.** TEM image (a) and powder X-ray diffraction pattern (b) of Sb_2_S_3_ nanoplates.


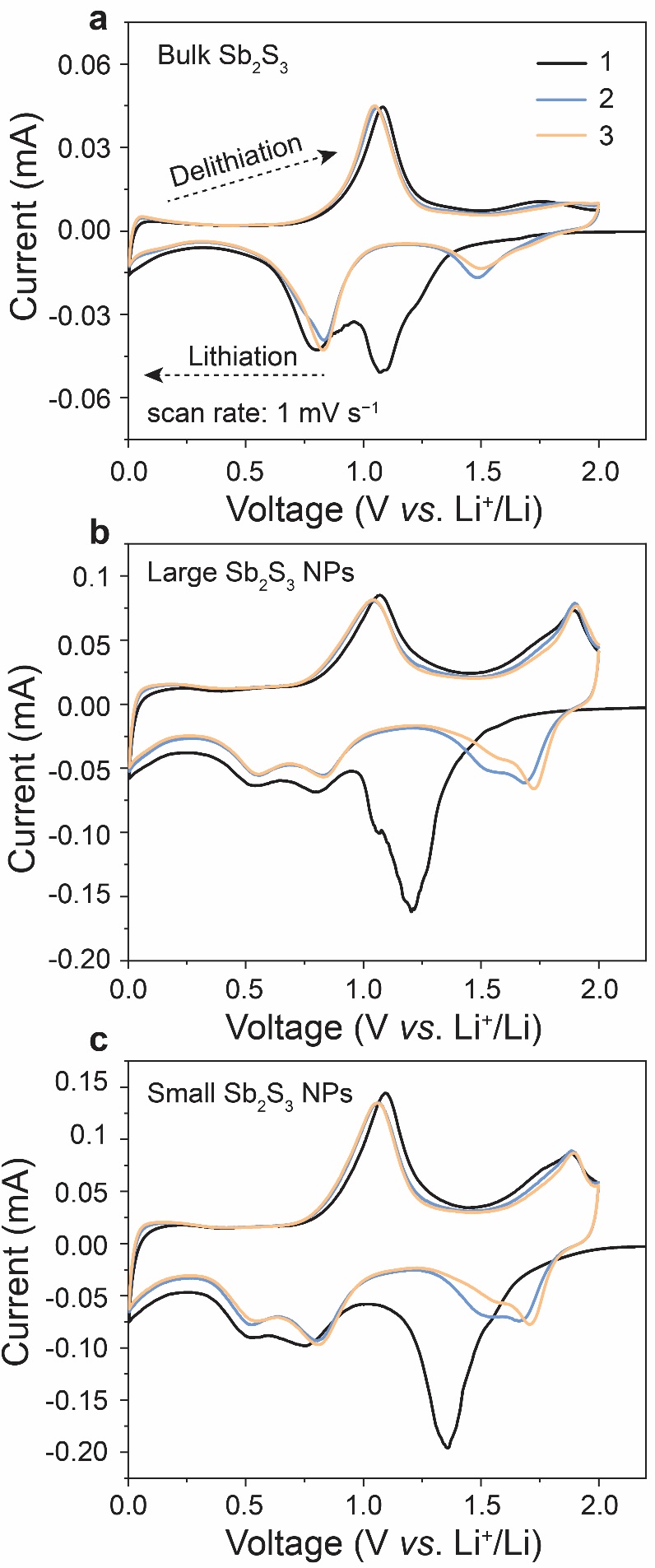


**Figure S8**. Cyclic voltammetry curves of bulk Sb_2_S_3_ particles (a), large (b) and small (c) Sb_2_S_3_ NPs measured in a half-cell configuration with metallic lithium at scan rate of 1 mV s^−1^.


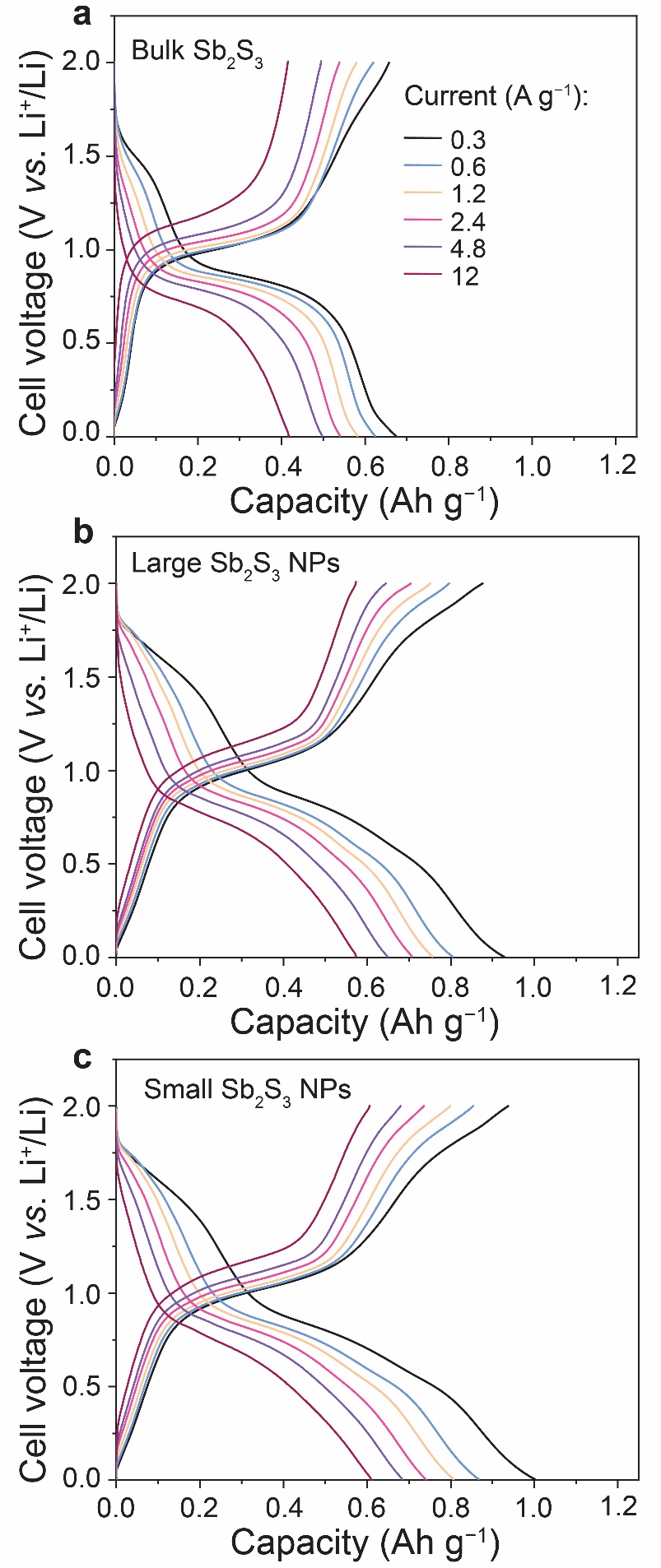


**Figure S9**. Galvanostatic charge-discharge curves of bulk Sb_2_S_3_ particles (a), large (b) and small (c) Sb_2_S_3_ NPs measured in a half-cell configuration with metallic lithium at different current densities.


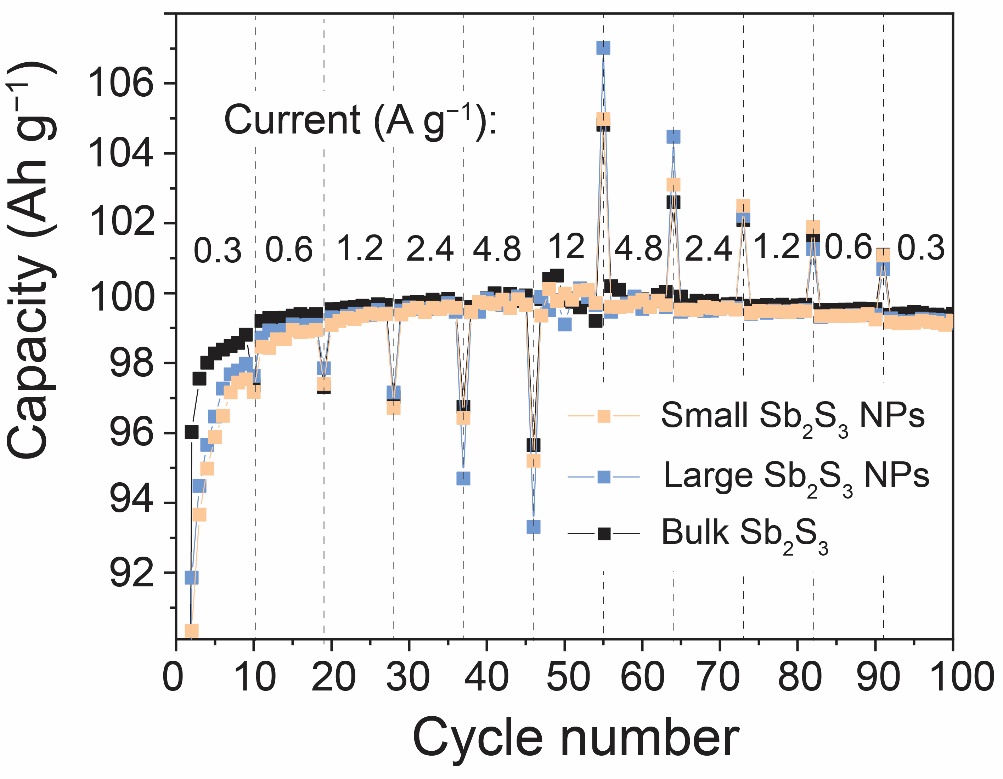


**Figure S10**. Coulombic efficiency of Sb_2_S_3_ electrodes cycled at different current densities of 0.3-12 A g^−1^ with lithium electrolyte (1M LiPF_6_ in EC/DMC) in a half-cell configuration.

**
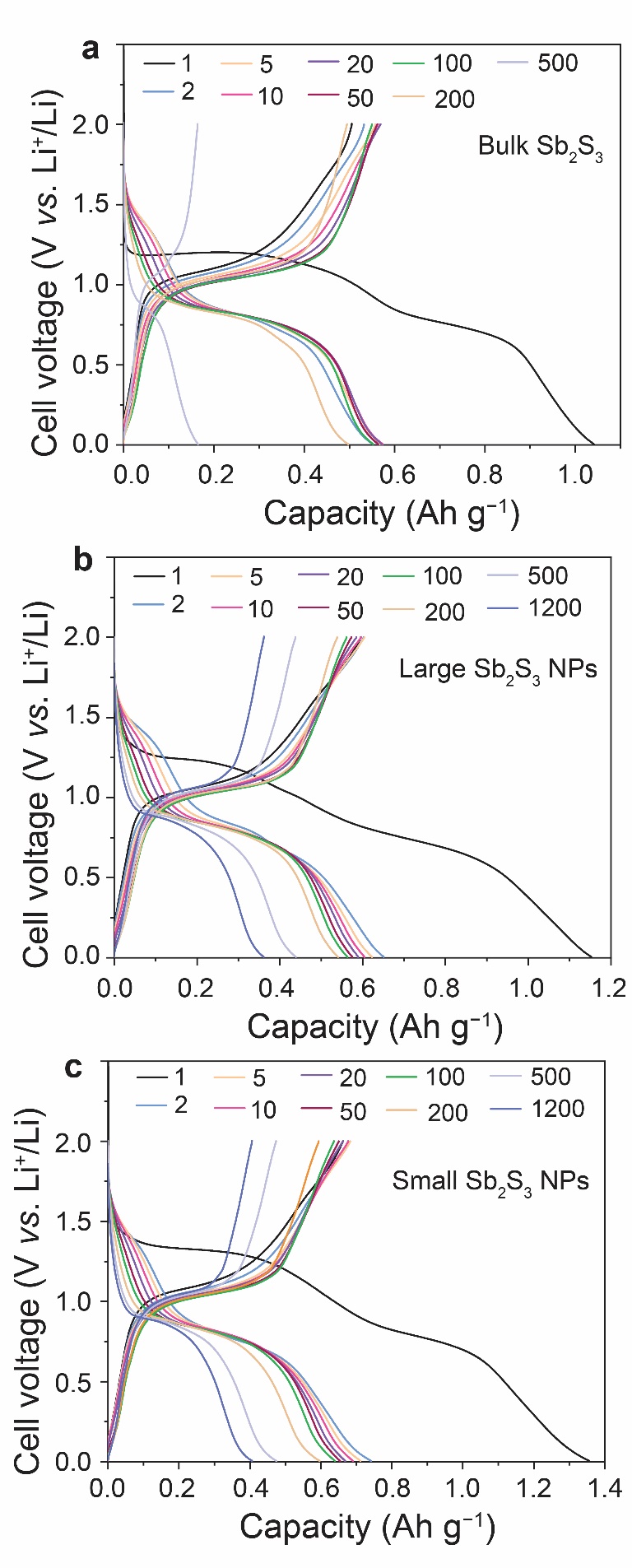
**

**Figure S11**. Galvanostatic charge-discharge curves of bulk Sb_2_S_3_ particles (a), large (b) and small (c) Sb_2_S_3_ NPs for different cycle numbers measured in a half-cell configuration with metallic lithium at current density of 2.4 A g^-1^.


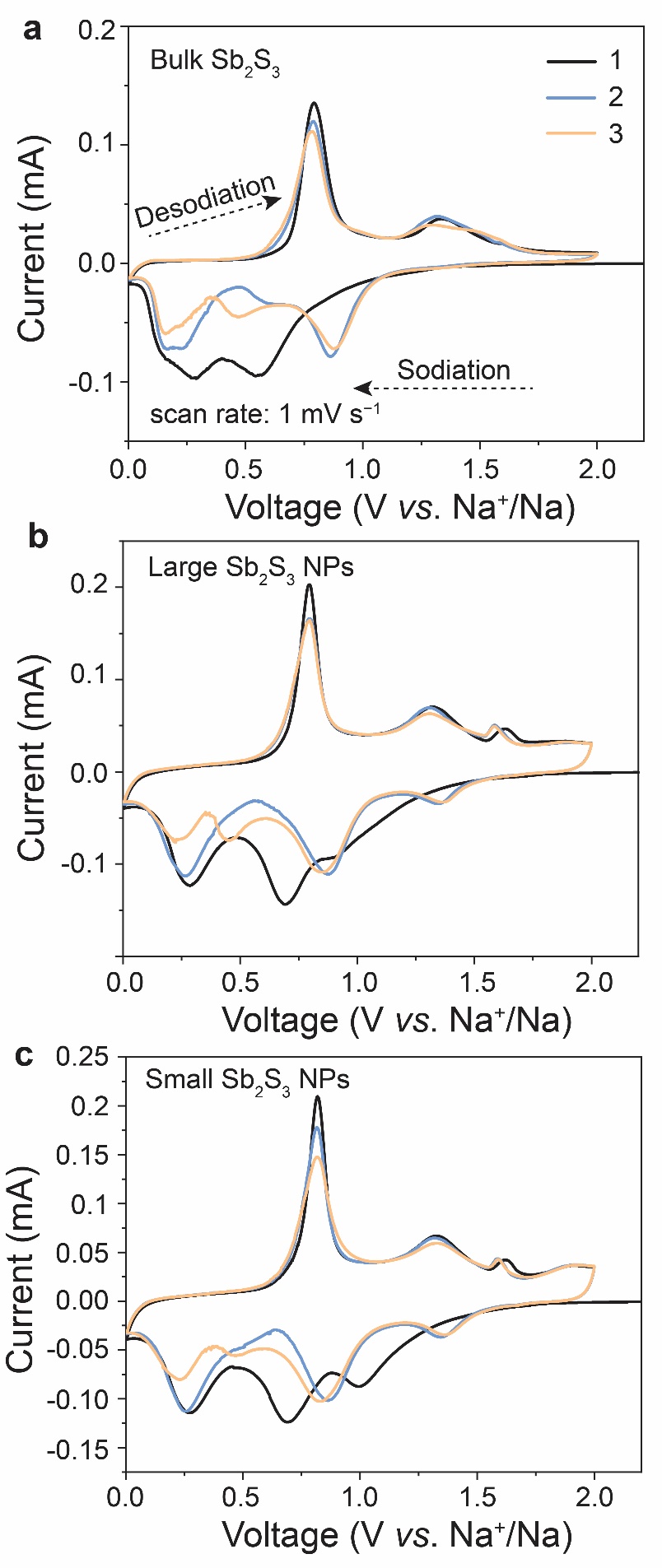


**Figure S12**. Cyclic voltammetry curves of bulk Sb_2_S_3_ particles (a), large (b) and small (c) Sb_2_S_3_ NPs measured in a half-cell configuration with metallic sodium at scan rate of 1 mV s^−1^.


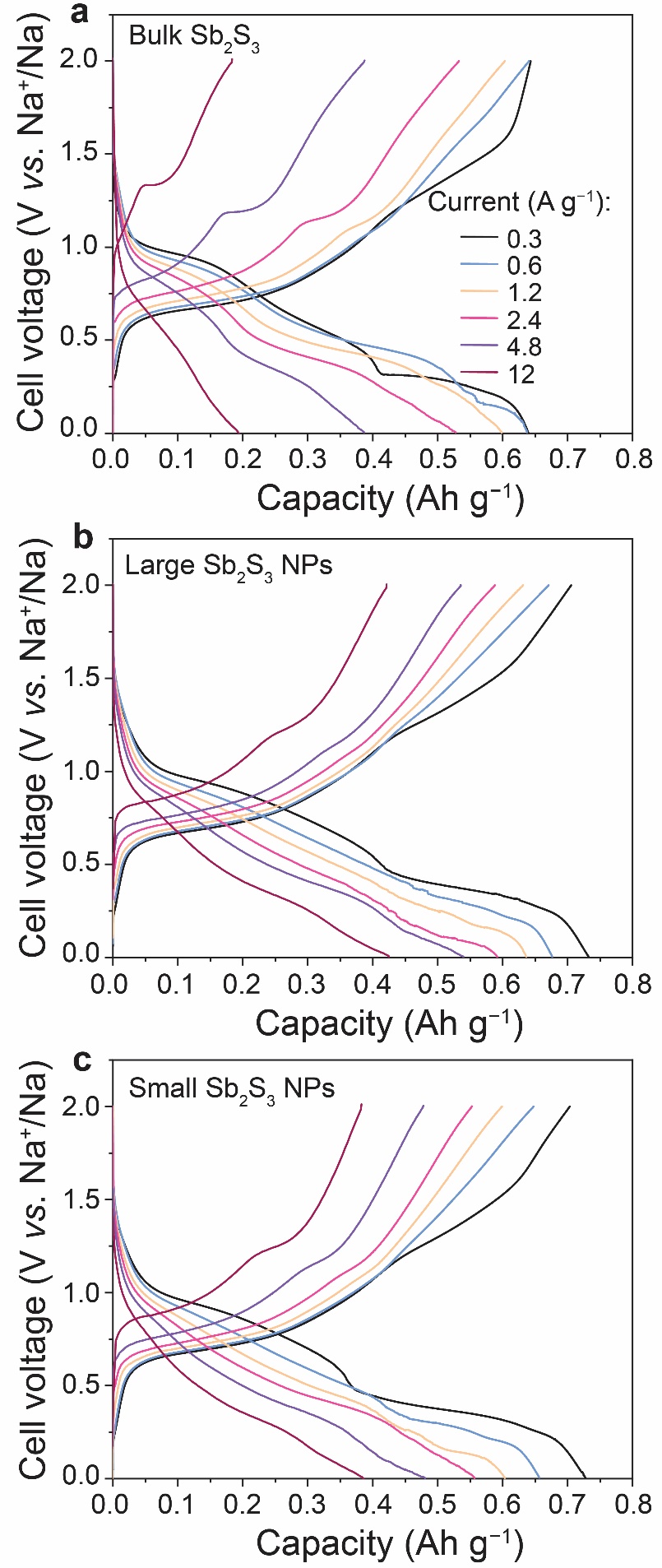


**Figure S13**. Galvanostatic charge-discharge curves of bulk Sb_2_S_3_ particles (a), large (b) and small (c) Sb_2_S_3_ NPs measured in a half-cell configuration with metallic sodium at different current densities.


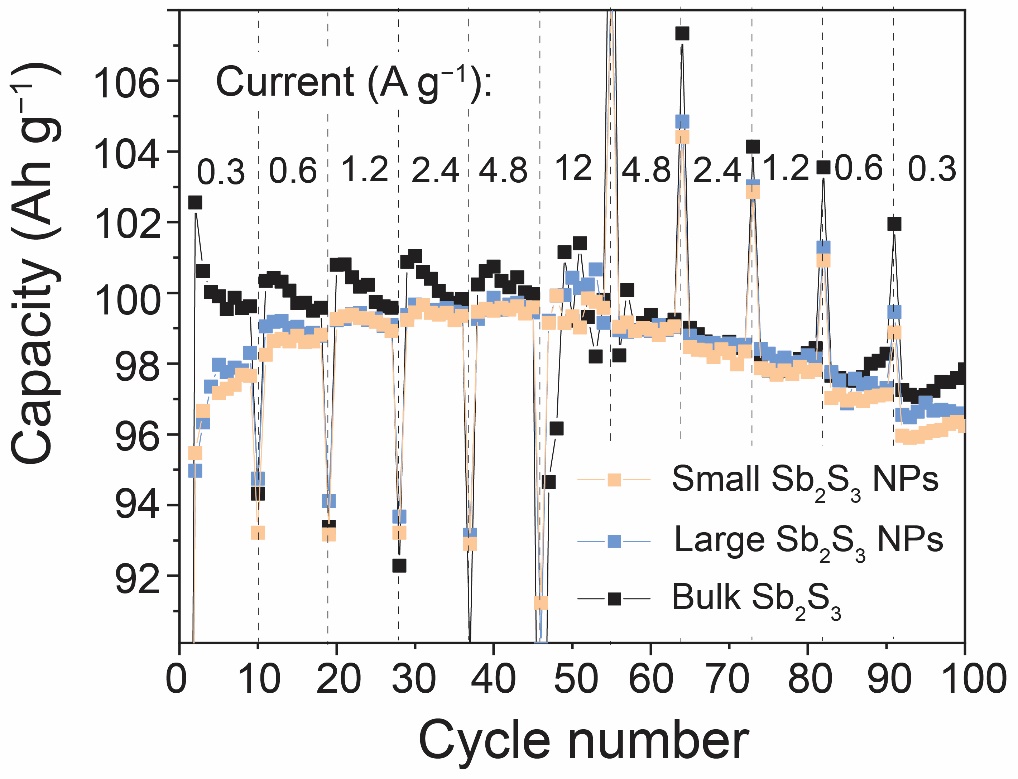


**Figure S14.** Coulombic efficiency of Sb_2_S_3_ electrodes cycled at different current densities of 0.3-12 A g^−1^ with sodium electrolyte (1M NaClO_4_ in PC) in a half-cell configuration.


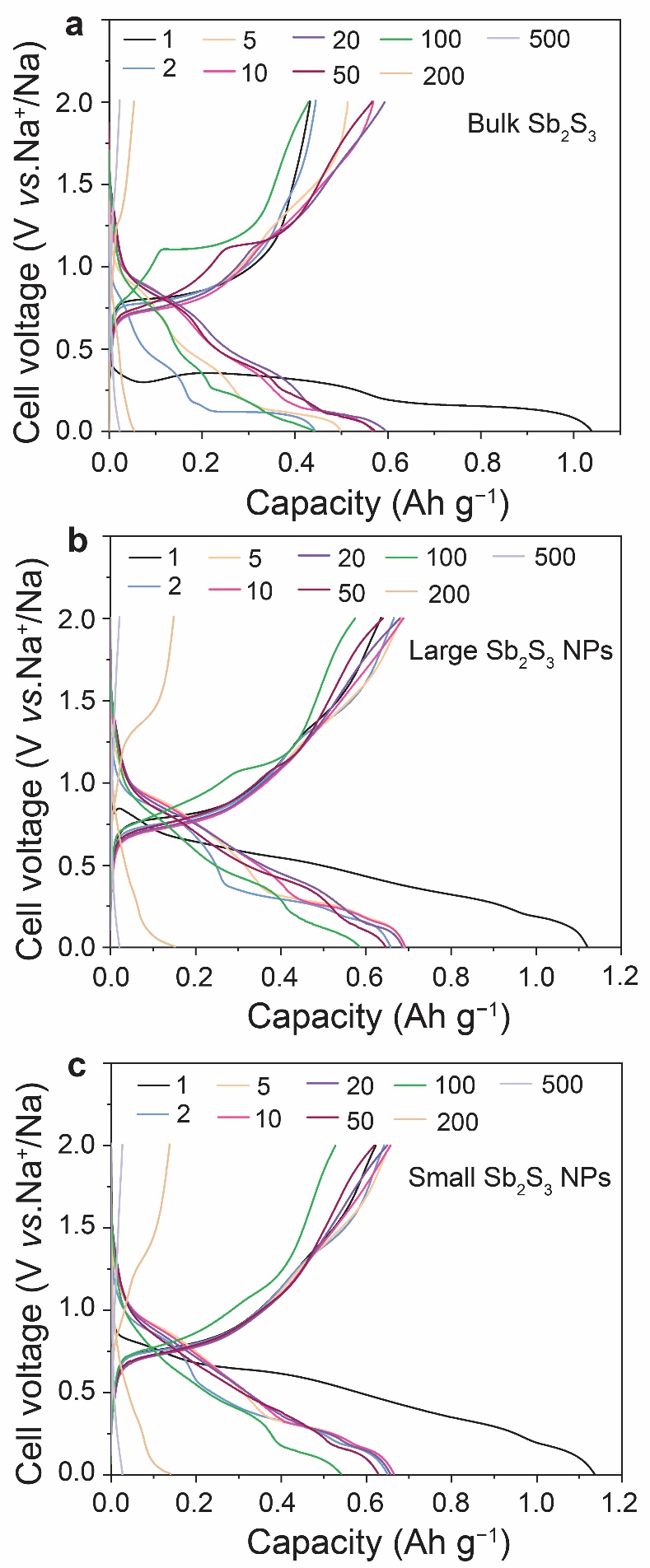


**Figure S15.** Galvanostatic charge-discharge curves of bulk Sb_2_S_3_ particles (a), large (b) and small (c) Sb_2_S_3_ NPs for different cycle numbers measured in a half-cell configuration with metallic sodium at current density of 2.4 A g^-1^.
